# Comparative Evaluation of Nutritional Quality and In Vitro Protein Digestibility in Selected Vegetable Soybean Genotypes at R6 and R8 Maturity

**DOI:** 10.3390/foods14142549

**Published:** 2025-07-21

**Authors:** Kanneboina Soujanya, T. Supraja, Aparna Kuna, Ramakrishnan M. Nair, S. Triveni, Kalenahalli Yogendra

**Affiliations:** 1 Department of Foods and Nutrition, PGRC, College of Community Science, PJTS Agricultural University, Rajendranagar, Hyderabad 500030, India; 2 MFPI—Quality Control Laboratory, Professor Jayashankar Telangana Agricultural University, Rajendranagar, Hyderabad 500030, India; 3 World Vegetable Center, South Asia, ICRISAT Campus, Patancheru, Hyderabad 502324, India; 4 Department of Agricultural Microbiology and Bioenergy, College of Agriculture, Hyderabad 500030, India; 5 Cell and Molecular Biology, ICRISAT, Patancheru, Hyderabad 502324, India

**Keywords:** R6 stage, R8 stage, vegetable soybean, nutritional quality, minerals, in vitro protein digestibility

## Abstract

The nutritional and quality characteristics of improved vegetable soybean genotypes were evaluated and compared with those of a grain-type soybean at the R6 (green maturity) and R8 (physiological maturity) stages. Significant variation (*p* < 0.05) was observed among genotypes for all measured traits. The overall quality parameters increased from the R6 (green maturity) stage to the R8 (physiological maturity) stage. Among the R6-stage genotypes, AVSB2001 recorded the highest contents of protein (15.30 ± 0.57 g/100 g), ash (2.31 ± 0.06 g/100 g), fat (8.05 ± 0.17 g/100 g), and calcium (140.78 ± 0.97 mg/100 g). The genotype Karune exhibited significantly higher levels of total sugars, non-reducing sugars, iron, and magnesium than the other entries. At the R8 stage, Swarna Vasundhara showed the highest protein content (39.23%), while AGS 447 recorded the highest values for fat, total sugars, in vitro protein digestibility, iron, copper, magnesium, and manganese. Notably, in vitro protein digestibility was lower across all genotypes at the R8 stage compared to the R6 stage. These findings suggest that selected vegetable soybean genotypes possess substantial nutritional value and can contribute meaningfully to meeting the recommended dietary allowance (RDA) across different age and occupational groups, underscoring this research’s potential public health impact. Based on stage-specific quality profiles, R6-stage genotypes may be better suited for fresh vegetables, whereas R8-stage genotypes can be utilized similarly to grain-type soybean for processing into products such as dhal, oil, flour, and other value-added foods.

## 1. Introduction

The consumption of fresh legumes such as common beans (*Phaseolus vulgaris*), peas (*Pisum sativum*), groundnuts (*Arachis hypogaea*), and pigeon peas (*Cajanus cajan*) is typical across many regions. These fresh green legumes are rich sources of vitamins, minerals, dietary fiber, and antioxidants. Among them, vegetable soybeans (*Edamame*) stands out for its high nutraceutical value and is considered one of the most nutrient-dense vegetable crops [1].

Soybeans, a crop of immense nutritional value, are generally classified into grain-type and vegetable-type based on their intended harvest stage and use [2,3]. Globally, there is a significant and growing demand for vegetable soybeans, highlighting the importance of this crop in the global food market [4,5]. While widely cultivated and consumed in East Asian countries such as China, Japan, and Korea, where it has long been valued as a snack or side dish, awareness and consumption in regions such as Africa, Europe, South Asia, and West Asia remain limited. However, due to its superior taste and nutritional properties, particularly its high protein content and health benefits, its cultivation and popularity have gradually expanded to other parts of the world [4,6,7].

Vegetable soybean is increasingly recognized as a functional food due to its high content of complete protein, dietary fiber, vitamins, minerals, isoflavones, and other bioactive compounds [8,9]. It is also a rich source of health-promoting polyunsaturated fatty acids, particularly linoleic and α-linolenic acids. These properties contribute to its potential in reducing the risk of various non-communicable diseases. Traditionally, vegetable soybeans are boiled in salted water, roasted, stir-fried, or added to stews, soups, rice dishes, salads, and desserts [4,10,11,12,13]. It is also used in innovative products such as green tofu, green milk, noodles, cookies, sweets, and snacks, and can serve as a substitute for green peas or lima beans in most recipes [14,15,16]. Given its versatility and nutritional value, it has considerable potential to enhance global dietary quality. Vegetable soybeans can be consumed fresh or processed into value-added products [17].

With the rising global demand for plant-based meat analogues, vegetable soybeans present an attractive raw material. Although soy and pea proteins are currently the primary bases for such products, conventional soy proteins often carry a strong beany flavor. In contrast, vegetable soybeans have a milder taste, making them a preferable choice for plant-based meat formulations [18]. Furthermore, its short crop cycle (65–75 days), low input requirement, and soil-enriching properties make it a highly profitable and sustainable crop. Under irrigated systems, it can be cultivated four to six times yearly [16,19]. In India, the successful use of grain soybeans as an intercrop to improve yields is an inspiring example, indicating that vegetable soybeans could be integrated into existing cropping systems [20].

Vegetable soybeans can be consumed in various forms—boiled, roasted, or combined with other vegetables. After the harvest season, the dried seeds are often used to prepare dhal, similar to traditional pulses like mung and black gram. There is an opportunity to develop value-added products from vegetable soybeans, such as roasted dhal or snacks. Internationally, dried vegetable soybean is a popular snack, with processors and consumers emphasizing its quality attributes considerably [21].

Despite its benefits, the vegetable soybean remains underutilized in India. Its acreage is minimal compared to grain-type soybeans, and it has not yet been adopted commercially in several regions, including Telangana, Jharkhand, Madhya Pradesh, Maharashtra, and Odisha. While the global market for vegetable soybean is growing, fueled by increased awareness and international trade, its acceptance in India remains slow [22,23]. A key limitation is the lack of improved, domestically developed *edamame* cultivars suitable for local agro-climatic conditions [22].

One of the key limitations hindering the widespread adoption and cultivation of vegetable soybean in India is the vulnerability of currently available cultivars—particularly Swarna Vasundhara and Karune—to yellow mosaic disease (YMD). This viral disease, transmitted primarily by whiteflies, severely impacts plant growth, yield, and pod quality, posing a significant threat to production. The high susceptibility of these cultivars has restricted the geographic expansion and scaling up of vegetable soybean cultivation in central and northern India regions, despite favorable climatic conditions. To address this constraint, the World Vegetable Center (WorldVeg) has made notable progress in breeding efforts by developing improved lines that exhibit strong resistance to YMD. These new lines were developed by introgressing resistance genes from SL958, a known YMD-resistant donor genotype [24]. Such genetic advancements not only improve crop resilience but also open up possibilities for expanding the cultivation of vegetable soybean into new regions, thereby enhancing the overall production potential. Although the vegetable soybean is not widely cultivated, its potential for improving nutritional security is immense [25]. The limited scale of production has resulted in a gap in research, particularly on its chemical and nutritional composition, which are key determinants of marketability and consumer acceptance [11].

Although India is among the world’s leading producers of grain soybeans, awareness, production, and utilization of vegetable soybeans are still in their infancy. A significant challenge is the lack of knowledge about its nutritional potential and health benefits. As consumer demand for plant-based foods continues to rise, vegetable soybean has the potential to gain popularity in India as a protein-rich food source [11,26]. Understanding its nutritional composition will benefit food industries, support biofortification efforts, and help combat malnutrition. Therefore, the present study aimed to evaluate and compare the nutritional composition of selected vegetable soybean genotypes at the R6 (green maturity) and R8 (physiological maturity) harvest stages.

## 2. Materials and Methods

### 2.1. Geographical Location and Details of Experimental Site

The investigation was conducted at the World Vegetable Center—South and Central Asia (WorldVeg–SCA) experimental farm at ICRISAT, Patancheru, Hyderabad, India. Eleven vegetable soybean improved lines/varieties (AVSB2001, AVSB2002, AVSB2004, AVSB2006, AVSB2007, AVSB2009, AVSB2012, AVSB2013, Swarna Vasundhara, Karune, and AGS447), along with one grain soybean variety (DSb34), were obtained from the World Vegetable Center, ICRISAT. Among these, nine vegetable soybean lines were developed explicitly to resist Yellow Mosaic Disease (YMD), a major limiting factor for expanding vegetable soybean cultivation in India. The genotypes were evaluated using a Randomized Block Design (RBD) with three replications. The experimental field was divided into three blocks, and within each block, all treatments were randomly assigned to plots. This randomization process was independently repeated for each block. All samples were harvested at two growth stages: R6 (when seeds are fully developed but still immature and green inside the pods) and R8 (full seed maturity) for subsequent evaluation. The samples were powdered, and the analysis was carried out in triplicate. Chemical analyses of the genotypes were performed at the Post Graduate and Research Center, PJTSAU, Hyderabad, India.

### 2.2. Sensory Evaluation

A semi-trained panel (n = 20) of members from the Post Graduate and Research Centre, Professor Jayashankar Telangana Agricultural University (PJTAU), conducted a comprehensive evaluation of the boiled vegetable soybean genotypes (R6 and R8 stage). They used a 9-point hedonic scale, 1 to 9, where: 1 = I dislike immensely (very bad) and 9 = I like extremely (excellent), to assess characteristics such as colour, texture, flavor, taste, mouthfeel, and overall acceptability. The samples were presented with three-digit code numbers in individual booths in the sensory evaluation lab. Panelists rinsed their mouths with water after testing each sample to neutralize the taste of the previous sample [27], ensuring the integrity of the evaluation process.

### 2.3. Chemical Analysis

Proximate composition of the selected genotypes at the R6 and R8 stages was analyzed following standard protocols. Moisture content was determined by drying a 5 g sample at 130 ± 3 °C for 2 h in a hot air oven, followed by cooling and weighing; results were expressed as g/100 g of sample [28]. Crude fat content was estimated using the Soxhlet extraction method [29]. Crude protein was measured by the Kjeldahl method [28], with nitrogen content multiplied by a conversion factor of 6.25 to express protein as g/100 g. Total ash content was determined using a standard protocol [30].

Crude fiber content was measured by sequential digestion, where samples were boiled with 1.25% dilute H_2_SO_4_, washed with water, then boiled again with 1.25% dilute NaOH, followed by a final wash with water. The residue remaining after digestion was recorded as crude fiber [31]. Total and available carbohydrate contents were calculated by difference.

Energy value (kcal/100 g) was calculated using the formula [32]

Energy (kcal/100 g) = [Protein (g) × 4] + [Carbohydrate (g) × 4] + [Fat (g) × 9].

Total dietary fiber (TDF) was determined based on enzymatic and gravimetric methods following a standard protocol [33]. Total sugars, reducing sugars, and non-reducing sugars were estimated according to the method described by Somogyi [34]. In vitro protein digestibility of the genotypes was assessed using the procedure prescribed by Schecterle and Pollak [35]. The sample was treated with pepsin (12.5 mg of pepsin in 50.0 mL of 0.1 N HCl at 37 °C for 3 h) followed by pancreatin (6.0 mg in 25.0 mL of phosphate buffer (pH 8.0)) for 24 h at 37 °C. The enzyme-treated sample was then analyzed for protein content using the Kjeldahl method as per AOAC 954.01—2010. For mineral analysis, samples were wet-digested using a microwave digester with nitric acid. Iron, calcium, zinc, sodium, potassium, copper, magnesium, and manganese were quantified by Inductively Coupled Plasma Atomic Absorption Spectrophotometry [36]. Phosphorus content was determined using the method described in [37].

The percentage Adequacy of the Recommended Dietary Allowance (RDA) of individual nutrients was calculated for women and men at different age groups, based on 100 g of selected vegetable soybean from the two studied stages. The RDA values for the respective nutrients were obtained from the ICMR-NIN guidelines.

### 2.4. Statistical Analysis

All experiments were conducted in replicates, and the results obtained were presented as the mean ± standard deviation (SD). The treatment means were compared for significant differences by one-way analysis of variance (ANOVA), and the means were separated using Duncan’s multiple range test at the 1% level using SPSS 22.0 (IBM, New York, NY, USA).

## 3. Results and Discussion

### 3.1. Sensory Evaluation of Vegetable Soybean Genotypes

**Figure 1:**
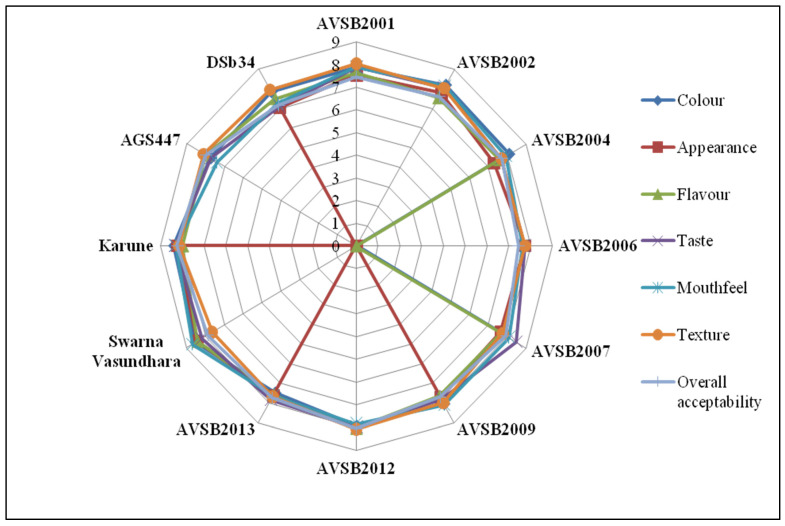
Mean sensory scores of vegetable soybean genotypes at the R6 stage.

**Figure 2:**
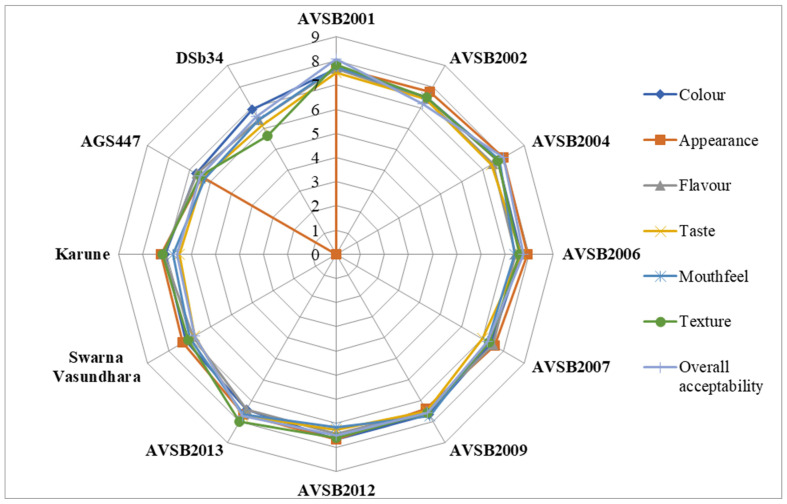
Mean sensory scores of vegetable soybean genotypes at the R8 stage.

In the present study, the sensory characteristics of boiled vegetable soybean genotypes were systematically evaluated at two critical maturity stages—R6 (immature green seed stage) and R8 (fully mature stage). A panel of 20 experienced and semi-trained experts, well-versed in the nuances of sensory evaluation, assessed key sensory attributes, including color, appearance, flavor, taste, texture, and overall acceptability, using a structured nine-point hedonic scale to ensure consistent and reliable evaluation across genotypes and stages. The analysis revealed significant differences in sensory appeal between genotypes and maturity stages. At the R6 stage, Karune and Swarna Vasundhara genotypes were rated highest regarding overall acceptability (Figure 1). Their vibrant green color, tender texture, and pleasant flavor profile likely contributed to their favorable scores. These qualities are critical for vegetable-type consumption, where visual appeal and tenderness are highly valued by consumers. Conversely, at the R8 stage, the genotypes AVSB2004 and AVSB2001 emerged as frontrunners in sensory evaluations, showcasing their potential for diverse culinary applications (Figure 2). Despite being harvested at full maturity, these genotypes retained a desirable flavor and acceptable texture after boiling, indicating their potential for use in dry bean or mature seed-based preparations where firmness and nutty flavor are more appreciated. The findings clearly demonstrate that sensory preferences are strongly influenced by the stage of maturity at harvest. Genotypes highly acceptable at the R6 stage may not maintain the same sensory appeal at R8 and vice versa. This stage-specific variation in consumer preference suggests that a single genotype may not fulfill consumer expectations across different end-use scenarios. Therefore, for product development and market targeting, it is crucial to consider both the genotype and the intended stage of consumption. This nuanced approach, as demonstrated by the suitability of different genotypes for different markets, enlightens us about the complexity of product development. For example, Karune and Swarna Vasundhara may be recommended for use in fresh edamame markets, whereas AVSB2004 and AVSB2001 may be better suited for mature soybean-based products. This also highlights the need for dual-purpose breeding strategies that consider not only agronomic traits but also consumer-driven sensory attributes aligned with harvest maturity stages.

### 3.2. Nutritional Composition of Vegetable Soybean Genotypes

Proximate composition (crude protein, crude lipid, ash, total carbohydrate, and crude fibre), vitamins, and mineral content of R6-stage and R8-stage vegetable soybean genotypes were analyzed, and the results are presented in Table 1, Table 2, Table 3, Table 4, Table 5, Table 6 and Table 7.

#### 3.2.1. Proximate Composition of Vegetable Soybean at the R6 and R8 Stage

Moisture and Dry Matter Content of Vegetable Soybean Genotypes: Moisture content is a critical quality attribute in food ingredients, directly affecting appearance, texture, and taste [37]. In the case of the vegetable soybean, moisture not only influences sensory properties but also serves as an indicator of pod maturity and marketability [38]. At the R6 stage (immature green seed stage), the moisture content of raw vegetable soybean genotypes ranged from 62.78% to 71.39%. Genotypes AVSB2004, Swarna Vasundhara, Karune, and AGS447 exhibited moisture levels that were 1.42%, 6.29%, 1.62%, and 8.21% higher, respectively, than the grain-type soybean (DSb34), while the remaining genotypes recorded lower moisture content (Table 1).

The moisture content values observed in these genotypes are consistent with previous reports [38,39,40], documenting moisture levels ranging from 62.19% to 72.90% in vegetable soybeans. High moisture content is a characteristic trait of vegetable soybean [41], contributing to its fresh and tender quality. However, this also makes the product highly perishable, necessitating cold storage solutions such as refrigeration and freezing to maintain postharvest quality and extend shelf life [40].

At the R8 stage (full seed maturity), the moisture content of the vegetable soybean genotypes dropped sharply, ranging between 5.35% (Karune) and 6.95% (AVSB2006) (Table 2). Genotypes AVSB2002, AVSB2006, AVSB2007, AVSB2009, AVSB2012, and Swarna Vasundhara recorded moisture content 0.43%, 0.77%, 0.12%, 0.07%, 0.21%, and 0.63% higher, respectively, than the grain soybean. In contrast, genotypes AVSB2001, AVSB2004, AVSB2013, Karune and AGS447 showed lower moisture values by 7.45%, 0.34%, 0.15%, 1.62%, and 0.90%, respectively.

**Figure 3:**
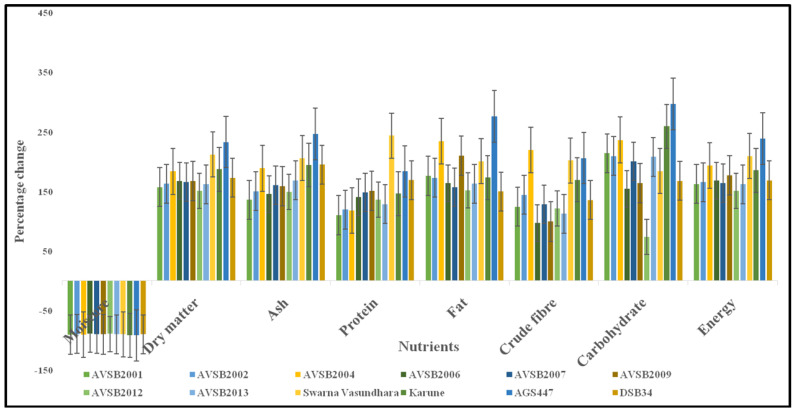
Percentage change in proximate composition of vegetable soybean genotypes from the R6 to R8 stage.

A substantial reduction in moisture content was observed from R6 to R8 across all genotypes. The percentage decrease in moisture content was as follows: AVSB2001 (90.60%), AVSB2002 (89.57%), AVSB2004 (90.72%), AVSB2006 (89.35%), AVSB2007 (89.94%), AVSB2009 (90.02%), AVSB2012 (89.52%), AVSB2013 (90.13%), Swarna Vasundhara (90.22%), Karune (92.01%), and AGS447 (91.82%) (Figure 3). This decline highlights the significant physiological and compositional changes during seed maturation. The moisture content observed at the R8 stage was lower than that reported for other legumes: 9–11.28% in peas [42], 6.25–11.60% in soybean [42], and 9.08–11.00 g/100 g (dry basis) in haricot bean [43].

Moisture content plays a key role in determining the storage stability, ease of dehulling, and overall processability of legume seeds. Understanding moisture dynamics is essential for food processors and researchers, as it impacts multiple processing stages—including drying, packaging, storage, cooking, and consumption [44]. The dry matter content of vegetable soybean genotypes showed significant variation at the 1% significance level. At the R6 stage, AVSB2012 recorded the highest dry matter content (37.21 g/100 g), while AGS447 showed the lowest (28.28 g/100 g). The dry matter content was inversely related to moisture content, confirming the impact of moisture variability among genotypes. During the R8 stage, dry matter content increased markedly, ranging from 93.14 g/100 g to 94.65 g/100 g. The highest dry matter content was observed in Karune, followed by AGS447 and AVSB2006. Compared to the R6 stage, the dry matter content of all genotypes increased by 151.03% to 232.96% at the R8 stage, reflecting advanced physiological maturity and water loss.

Macronutrients play a vital role in human nutrition by providing energy, maintaining essential body structures, supporting physiological functions, and preventing diseases. This study analyzed key macronutrients, including protein, oil content, crude fiber, dietary fiber, and total carbohydrates, in vegetable soybean genotypes at the R6 and R8 developmental stages, with results summarized in Table 1 and Table 2. Vegetable soybean was confirmed as a rich protein source, with protein content at the R6 stage ranging from 11.42 g/100 g (Swarna Vasundhara) to 15.30 g/100 g (AVSB2001) on a fresh weight basis. At the R8 stage, protein concentration significantly increased across all genotypes, attributed mainly to moisture loss during seed maturation, reaching levels between 30.24 g/100 g (AVSB2004) and 39.23 g/100 g (Swarna Vasundhara). These findings align with previous studies reporting 32.2% to 45.7% of seed protein content in Indian and black soybean varieties [45,46]. It is well-established that genetic factors, environmental conditions, and fertilizer application considerably influence soybean protein content [47].

Legumes generally contain high levels of dietary protein (20–40 g/100 g dry matter), approximately double that found in cereals [48]. However, legumes tend to be limited in sulfur-containing amino acids such as cystine and methionine, making them incomplete proteins. Conversely, cereals typically have low lysine and tryptophan levels. Therefore, combining legumes with cereals can improve the overall dietary amino acid profile, enhancing protein quality [47]. Protein is a crucial macronutrient and a primary nitrogen source in the human diet. Its functional properties also make it an essential ingredient in food formulations. With its high protein content and quality, vegetable soybean represents an excellent dietary option for vegans and vegetarians [7,48]. Moreover, high-protein soybean varieties can contribute to addressing protein–energy malnutrition and support tissue growth, maintenance, repair, and immune function [49,50].

Compared to grain-type soybean (DSb34) at the R6 stage, most vegetable soybean genotypes exhibited higher protein content, except AGS447 and Swarna Vasundhara. At the R8 stage, protein content in AVSB2006, AVSB2007, Swarna Vasundhara, Karune, and AGS447 exceeded that of DSb34 by 2.11%, 14.66%, 27.58%, 15.65%, and 1.28%, respectively. In contrast, AVSB2001, AVSB2002, AVSB2004, AVSB2009, AVSB2012, and AVSB2013 showed lower protein levels than DSb34 by 25.92%, 35.37%, 40.36%, 1.88%, 6.12%, and 9.22%, respectively. Across all genotypes, a significant increase in protein percentage was observed from the R6 to the R8 stage. The percentage increase ranged from 110.13% in AVSB2001 to 243.52% in Swarna Vasundhara, indicating ongoing protein accumulation and seed maturation processes [51]. Saldivar et al. [52] also reported that protein content in vegetable soybean genotypes increased from 5 to 10–11 weeks after flowering (R6–R8 stages). From a nutritional standpoint, consuming 100 g of vegetable soybean can contribute substantially to daily protein requirements. At the R6 stage, this amount fulfills approximately 24.83 to 33.26% of the Estimated Average Requirement (EAR) for protein in females, and 20.39 to 27.32% in males. At the R8 stage, the contribution significantly increases, meeting 67.17 to 85.28% of EAR in females and 55.17 to 71.11% in males. These gender-specific values underscore the nutritional potential of vegetable soybeans, particularly at full maturity, and support their inclusion in protein-enriched dietary planning, especially in regions where plant-based protein sources are essential [53].

Protein accumulation during seed development follows a dynamic pattern. Previous research indicates an initial decline in protein levels by 2–6% during the first 3–5 weeks after flowering, followed by a gradual increase toward maturity, correlating with oil and starch synthesis dynamics [52]. The quality of soybean protein is comparable to animal protein and meets the WHO/FAO/UNU standards for plant protein adequacy [54]. The protein content observed in vegetable soybean at the R6 stage further highlights its nutritional value compared to common vegetables, reinforcing its importance as a functional and economic component in food products [55].

Fat is a critical macronutrient that serves as a concentrated source of metabolic energy and facilitates the absorption of fat-soluble vitamins (A, D, E, and K) and bioactive compounds such as carotenoids [17]. The present study observed a statistically significant variation (*p* < 0.01) in the crude oil content among vegetable soybean genotypes at both R6 and R8 developmental stages. At the R6 stage, crude oil content ranged from 5.95 g/100 g (AGS447) to 8.06 g/100 g (AVSB2013), with most genotypes exhibiting intermediate values: AVSB2001 (8.05), AVSB2002 (6.90), AVSB2004 (6.47), AVSB2006 (7.46), AVSB2007 (7.83), AVSB2009 (7.04), AVSB2012 (7.85), Swarna Vasundhara (6.85), Karune (6.89), and DSb34 (7.41 g/100 g). At the R8 stage, oil content increased markedly in all genotypes, ranging from 18.52 g/100 g in DSb34 to 22.38 g/100 g in AGS447. This increase reflects the accumulation of lipids during seed maturation, a typical physiological phenomenon in soybean development.

Genetic factors, environmental conditions, and geographical location influence the variation in oil content among genotypes. Soybean oil is nutritionally valued for its high content of polyunsaturated fatty acids (PUFAs), particularly essential fatty acids such as linoleic acid and alpha-linolenic acid. These compounds have been reported to confer cardioprotective, anticancer, and anti-obesity effects [49]. From a dietary perspective, vegetable soybeans with high protein and comparatively lower oil content at the R6 stage can be a desirable snack option for individuals following high-protein, low-fat diets [46,56]. The U.S. Food and Drug Administration (FDA) recognized soybean protein’s health benefits, stating in 1999 that a daily intake of 25 g may help reduce the risk of coronary heart disease [57].

A notable increase in oil content was observed from R6 to R8 across all genotypes. The percentage increase was genotype-dependent and ranged as follows: AVSB2001 (176.02%), AVSB2002 (172.75%), AVSB2004 (234.62%), AVSB2006 (163.54%), AVSB2007 (156.57%), AVSB2009 (209.80%), AVSB2012 (151.72%), AVSB2013 (162.78%), Swarna Vasundhara (200.73%), Karune (173.15%), AGS447 (276.13%), and DSb34 (149.93%). Among R6 genotypes, AVSB2001, AVSB2006, AVSB2007, AVSB2009, AVSB2012, AVSB2013, and DSb34 had significantly higher oil content than the others. By contrast, at the R8 stage, all vegetable soybean genotypes demonstrated substantially higher fat content than the grain-type soybean (DSb34), reinforcing the nutritional enhancement with seed maturation.

Ash is the inorganic residue remaining after the complete combustion or oxidation of organic matter in food. It represents the total mineral content and is a critical component of proximate analysis used in nutritional evaluation [58,59]. Ash analysis provides a preliminary estimate of the total mineral load and is a basis for further elemental characterization. In the present study, the total ash content of vegetable soybean genotypes at the R6 stage ranged from 1.57 g/100 g to 2.31 g/100 g (fresh weight basis). At the R8 stage, a significant increase in ash content was observed across all genotypes. The values ranged from 4.98 g/100 g (AVSB2006) to 5.81 g/100 g (DSb34), with individual genotype values as follows: AVSB2001 (5.45), AVSB2002 (5.28), AVSB2004 (5.63), AVSB2006 (4.98), AVSB2007 (5.29), AVSB2009 (5.23), AVSB2012 (5.56), AVSB2013 (5.61), Swarna Vasundhara (5.08), Karune (5.74), AGS447 (5.44), and DSb34 (5.81 g/100 g).

Comparative analysis between stages revealed that, except for AVSB2004, Swarna Vasundhara, Karune, and AGS447, all genotypes had higher ash content at R6 than grain-type soybean. However, when comparing vegetable soybean genotypes to the grain-type control (DSb34) at the R8 stage, all vegetable soybeans showed significantly higher ash content. The increase in ash content from R6 to R8 was substantial, ranging between 135.93% and 246.49%, indicating progressive mineral concentration as moisture content declined during seed maturation at the R8 stage.

Though nutritionally limited, crude fiber content plays an essential physiological role by providing bulk that supports peristaltic movement in the gastrointestinal tract [60]. It quantifies indigestible plant components such as cellulose, hemicellulose (pentosans), and lignin. Significant differences (*p* < 0.01) were observed among genotypes at both stages in crude fiber content. At R6, crude fiber ranged from 2.30 g/100 g (AGS447) to 3.27 g/100 g (AVSB2009). At R8, values increased, ranging from 6.25 g/100 g (AVSB2013) to 7.73 g/100 g (AVSB2004). Notably, at R6, only four genotypes, i.e., AVSB2006, AVSB2007, AVSB2009, and AVSB2012, exhibited higher crude fiber content than the grain-type control (DSb34). In contrast, at R8, only AVSB2004 and Swarna Vasundhara surpassed the DSb34 fiber content.

A significant increase in crude fiber content was recorded across all genotypes from R6 to R8, attributable to the physiological maturation process. The percentage increases were as follows: AVSB2001 (124.32%), AVSB2002 (144.16%), AVSB2004 (219.42%), AVSB2006 (96.85%), AVSB2007 (127.92%), AVSB2009 (99.39%), AVSB2012 (121.12%), AVSB2013 (112.58%), Swarna Vasundhara (202.05%), Karune (169.47%), AGS447 (206.09%), and DSb34 (135.33%). These results highlight the nutritional enhancement of vegetable soybeans during maturation, with significant increases in mineral and crude fiber content, reinforcing their role as a functional and nutrient-dense legume crop. Kambhampati et al. [61] reported that metabolite levels of primary intermediates, such as amino acids, sugars, and organic acids, decline throughout development, while the storage components that include RFOs (raffinose family oligosaccharides), lipids, and proteins increase.

Carbohydrates are a primary energy source in plant-based foods and serve as dietary fiber. They typically account for 50–80% of the dry weight of plant foods. In nutritional analysis, the total carbohydrate content of a sample is calculated by difference, subtracting the sum of moisture, crude protein, total fat, and ash content from the total weight of the food sample [62]. In the present study, total carbohydrate content in vegetable soybean genotypes was estimated by the difference method, and the results are presented in Table 3 and Table 4. At the R6 stage, total carbohydrate content varied significantly among genotypes (*p* < 0.01). AVSB2012 exhibited the highest carbohydrate content (12.41 g/100 g), while AGS447 showed the lowest (8.47 g/100 g). These results were consistent with the findings of [63], who reported carbohydrate levels ranging from 7.70 to 11.08 g/100 g (fresh weight basis) in vegetable soybeans.

At the R8 stage, a marked increase in total carbohydrate content was observed, ranging from 26.31 g/100 g (AVSB2006) to 38.25 g/100 g (AVSB2002). During R6, most genotypes had lower carbohydrate levels compared to the grain-type control (DSb34), except for AVSB2002 (8.23% higher), AVSB2009 (3.23%), and AVSB2013 (8.67%). However, at R8, all genotypes except AVSB2006, AVSB2007, and Karune recorded significantly higher carbohydrate content than DSb34.

The percentage increase in total carbohydrate content from the R6 to R8 stage was substantial across genotypes, with AVSB2001, AVSB2002, AVSB2004, AVSB2006, AVSB2007, AVSB2009, AVSB2012, AVSB2013, Swarna Vasundhara, Karune, AGS447, and DSb34 showing increases of 214.14%, 209.47%, 236.39%, 154.20%, 200.49%, 164.04%, 73.33%, 208.16%, 184.19%, 259.14%, 297.05%, and 167.77%, respectively. At the R8 stage, the major carbohydrate fraction in soybeans consists of oligosaccharides, which bypass digestion in the upper gastrointestinal tract and reach the colon, where they promote the growth of beneficial gut microbiota such as *Bifidobacterium* spp. [64].

Energy content, a function of protein, fat, and carbohydrate levels, also varied significantly across genotypes. At the R6 stage, total energy values ranged from 137.94 kcal/100 g to 179.21 kcal/100 g. AVSB2012 showed the highest energy value, likely due to its elevated protein, lipid, and carbohydrate content. At the R8 stage, AGS447 had the highest energy value, while DSb34 exhibited the lowest. Except for Swarna Vasundhara, Karune, and AGS447, all vegetable soybean genotypes had higher energy content than the grain-type control (DSb34). The relatively lower energy content in Swarna Vasundhara, Karune, and AGS447 may be attributed to their higher moisture content, which is inversely related to the concentration of dry matter components such as protein, fat, fiber, and carbohydrates.

Energy content increased significantly from the R6 to R8 stages across all genotypes. The percentage increases were as follows: AVSB2001 (162.59%), AVSB2002 (165.53%), AVSB2004 (193.77%), AVSB2006 (167.86%), AVSB2007 (163.89%), AVSB2009 (176.83%), AVSB2012 (151.22%), AVSB2013 (161.90%), Swarna Vasundhara (209.50%), Karune (185.55%), AGS447 (238.40%), and DSb34 (168.45%). The study demonstrated that all vegetable soybean genotypes experienced significant increases in protein, ash, crude fiber, lipid, total carbohydrate, and energy content as they matured from the R6 to the R8 stage. These enhancements are associated with physiological development and biochemical accumulation of macronutrients during seed maturation. It is also evident that varietal differences, environmental conditions, and geographical location significantly influence vegetable soybean genotypes’ nutritional and quality profiles [65].

**Table 1:** Proximate composition of vegetable soybean genotypes at the R6 stage (g/100 g).

Genotypes	Moisture	Dry Matter	Ash	Protein	Fat	Crude Fibre	Carbohydrate	Energy (kcal)
AVSB2001	63.44 ± 1.19 ^ab^	36.55 ± 1.19 ^ef^	2.31 ± 0.06 ^e^	15.30 ± 0.57 ^f^	8.05 ± 0.17 ^g^	2.96 ± 0.16 ^de^	10.89 ± 0.53 ^cde^	177.25 ± 0.52 ^f^
AVSB2002	64.52 ± 0.23 ^bcd^	35.47 ± 2.16 ^def^	2.11 ± 0.16 ^d^	14.10 ± 0.30 ^cde^	6.90 ± 0.37 ^bcd^	2.74 ± 0.12 ^bcd^	12.36 ± 0.13 ^ef^	167.99 ± 0.98 ^de^
AVSB2004	66.91 ± 0.49 ^e^	33.08 ± 0.49 ^c^	1.95 ± 0.06 ^b^	13.89 ± 0.08 ^cd^	6.47 ± 0.32 ^ab^	2.42 ± 0.27 ^ab^	10.77 ± 0.34 ^cde^	156.88 ± 0.29 ^c^
AVSB2006	65.24 ± 0.84 ^cd^	34.76 ± 0.85 ^de^	2.03 ± 0.06 ^bcd^	14.92 ± 0.09 ^def^	7.46 ± 0.43 ^ef^	3.18 ± 0.10 ^e^	10.35 ± 0.44 ^cd^	168.21 ± 0.59 ^de^
AVSB2007	64.83 ± 0.19 ^bcd^	35.17 ± 0.19 ^def^	2.03 ± 0.04 ^bcd^	15.13 ± 0.31 ^ef^	7.83 ± 0.41 ^fg^	3.08 ± 0.08 ^e^	10.17 ± 0.05 ^bcd^	171.74 ± 0.27 ^def^
AVSB2009	65.05 ± 0.44 ^bcd^	34.95 ± 0.44 ^de^	2.02 ± 0.02 ^bcd^	14.08 ± 0.05 ^cde^	7.04 ± 0.15 ^cde^	3.27 ± 0.14 ^e^	11.79 ± 0.37 ^ef^	166.94 ± 0.95 ^d^
AVSB2012	62.78 ± 0.31 ^a^	37.21 ± 0.31 ^f^	2.23 ± 0.05 ^e^	14.71 ± 0.28 ^def^	7.85 ± 0.41 ^fg^	3.03 ± 0.09 ^de^	12.41 ± 0.45 ^ef^	179.21 ± 0.39 ^f^
AVSB2013	64.27 ± 0.49 ^abc^	35.73 ± 0.49 ^ef^	2.09 ± 0.04 ^cd^	15.04 ± 0.34 ^def^	8.06 ± 0.28 ^g^	2.94 ± 0.14 ^cde^	10.54 ± 0.38 ^cde^	174.87 ± 0.93 ^ef^
Swarna Vasundhara	70.12 ± 0.27 ^f^	29.87 ± 0.27 ^b^	1.66 ± 0.05 ^a^	11.42 ± 0.17 ^a^	6.85 ± 0.19 ^bcd^	2.44 ± 0.31 ^ab^	9.93 ± 0.21 ^bc^	147.09 ± 0.28 ^b^
Karune	67.04 ± 0.96 ^e^	32.95 ± 0.96 ^c^	1.95 ± 0.05 ^b^	15.29 ± 0.92 ^f^	6.89 ± 0.12 ^bc^	2.62 ± 0.12 ^abc^	9.03 ± 1.76 ^ab^	157.48 ± 3.64 ^c^
AGS447	71.39 ± 0.62 ^f^	28.28 ± 0.44 ^a^	1.57 ± 0.03 ^a^	12.61 ± 0.04 ^b^	5.95 ± 0.27 ^a^	2.30 ± 0.19 ^a^	8.47 ± 0.07 ^a^	137.94 ± 3.47 ^a^
DSb34	65.97 ± 0.15 ^de^	34.28 ± 0.15 ^cd^	1.97 ± 0.01 ^ab^	13.23 ± 0.26 ^bc^	7.41 ± 0.29 ^def^	3.00 ± 0.23 ^de^	11.42 ± 0.12 ^def^	165.25 ± 1.32 ^d^
F value	26.28	28.17	30.80	14.66	11.05	9.59	8.73	25.66
*p* value	0.00 **	0.00 **	0.00 **	0.00 **	0.00 **	0.00 **	0.00 **	0.00 **

**Note:** The values are the mean ± SD of (*n* = 3) replications. ** Significant at 1%. Values with a different superscript in the same column are significantly different (*p* ≤ 0.05).

**Table 2:** Proximate composition of vegetable soybean genotypes at the R8 stage (g/100 g).

Genotypes	Moisture	Dry Matter	Ash	Protein	Fat	Crude Fibre	Carbohydrate	Energy (kcal)
AVSB2001	5.96 ± 0.22 ^abc^	94.04 ± 0.22 ^bcd^	5.45 ± 0.06 ^c^	32.15 ± 1.83 ^a^	22.22 ± 0.22 ^f^	6.64 ± 0.34 ^abc^	34.21 ± 1.86 ^cde^	465.45 ± 0.89 ^g^
AVSB2002	6.73 ± 0.36 ^cd^	93.26 ± 0.36 ^ab^	5.28 ± 0.15 ^b^	30.90 ± 1.15 ^a^	18.82 ± 0.16 ^ab^	6.69 ± 0.14 ^bc^	38.25 ± 0.88 ^e^	446.07 ± 1.00 ^ab^
AVSB2004	6.21 ± 0.08 ^bcd^	93.79 ± 0.08 ^abc^	5.63 ± 0.11 ^de^	30.24 ± 0.77 ^a^	21.65 ± 0.03 ^ef^	7.73 ± 0.28 ^e^	36.23 ± 0.83 ^de^	460.87 ± 0.28 ^f^
AVSB2006	6.95 ± 0.43 ^d^	93.05 ± 0.43 ^a^	4.98 ± 0.06 ^a^	35.86 ± 0.97 ^bcd^	19.66 ± 0.43 ^bc^	6.26 ± 0.03 ^a^	26.31 ± 0.64 ^a^	450.57 ± 2.56 ^bc^
AVSB2007	6.52 ± 0.03 ^bcd^	93.48 ± 0.03 ^abc^	5.29 ± 0.08 ^b^	37.52 ± 1.68 ^cde^	20.09 ± 0.32 ^c^	7.02 ± 0.32 ^cd^	30.56 ± 1.93 ^bc^	453.22 ± 1.46 ^cd^
AVSB2009	6.49 ± 0.22 ^bcd^	93.51 ± 0.22 ^abc^	5.23 ± 0.06 ^b^	35.33 ± 1.17 ^bc^	21.81 ± 0.85 ^ef^	6.52 ± 0.34 ^ab^	31.13 ± 1.80 ^bc^	462.14 ± 0.59 ^fg^
AVSB2012	6.58 ± 0.73 ^bcd^	93.41 ± 0.73 ^abc^	5.56 ± 0.06 ^cd^	34.77 ± 1.37 ^b^	19.76 ± 0.78 ^bc^	6.70 ± 0.26 ^bc^	33.31 ± 1.32 ^cd^	450.22 ± 1.22 ^bc^
AVSB2013	6.34 ± 0.23 ^bcd^	93.63 ± 0.23 ^abc^	5.61 ± 0.11 ^de^	34.36 ± 0.82 ^b^	21.18 ± 0.81 ^de^	6.25 ± 0.13 ^a^	32.48 ± 0.89 ^bcd^	457.99 ± 0.45 ^ef^
Swarna Vasundhara	6.86 ± 0.98 ^d^	93.14 ± 0.97 ^a^	5.08 ± 0.04 ^a^	39.23 ± 0.90 ^e^	20.60 ± 0.38 ^cd^	7.37 ± 0.33 ^de^	28.22 ± 1.50 ^ab^	455.25 ± 3.24 ^de^
Karune	5.35 ± 0.05 ^a^	94.65 ± 0.05 ^d^	5.74 ± 0.04 ^ef^	37.65 ± 1.40 ^bcd^	18.82 ± 0.23 ^ab^	7.06 ± 0.16 ^cd^	32.43 ± 1.25 ^bcd^	449.69 ± 0.91 ^bc^
AGS447	5.84 ± 0.13 ^ab^	94.16 ± 0.13 ^cd^	5.44 ± 0.02 ^c^	35.75 ± 0.98 ^d^	22.38 ± 0.65 ^f^	7.04 ± 0.05 ^cd^	33.63 ± 1.35 ^bc^	466.79 ± 0.26 ^h^
DSb34	6.44 ± 0.35 ^bcd^	93.56 ± 0.35 ^abc^	5.81 ± 0.04 ^f^	35.58 ± 0.65 ^bcd^	18.52 ± 0.48 ^a^	7.06 ± 0.21 ^cd^	30.58 ± 1.30 ^cd^	443.62 ± 0.86 ^a^
F value	3.58	3.58	32.85	15.36	21.21	10.87	6.02	26.35
*p* value	0.00 **	0.00 **	0.00 **	0.00 **	0.00 **	0.00 **	0.00 **	0.00 **

**Note:** The values are presented as the mean ± SD of (*n* = 3) replications. ** Significant at 1%. Values with a different superscript in the same column are significantly different (*p* ≤ 0.05).

#### 3.2.2. Dietary Fiber, Sugars, and IVPD of Vegetable Soybean Genotypes at R6 and R8 Stage

Available Carbohydrates: Available carbohydrates refer to the sum of digestible carbohydrates, predominantly free sugars and starch, which are rapidly hydrolyzed and absorbed in the human small intestine, thereby serving as glucogenic substrates. This classification corresponds to the term “glycemic carbohydrates” [66]. At the R6 developmental stage, the available carbohydrate content in vegetable soybean genotypes ranged from 3.40 to 6.75 g/100 g (fresh weight basis). The lowest concentration was observed in AGS447, likely due to its relatively higher moisture content, which dilutes the concentration of dry matter constituents. At the R8 stage, available carbohydrate content increased substantially across genotypes, ranging from 7.61 g/100 g (Swarna Vasundhara) to 18.80 g/100 g (AVSB2001). The percentage increase in available carbohydrate content from R6 to R8 was significant, with AVSB2001, AVSB2002, AVSB2004, AVSB2006, AVSB2007, AVSB2009, AVSB2012, AVSB2013, Swarna Vasundhara, Karune, AGS447, and DSb34 exhibiting respective increases of 178.19%, 196.06%, 246.59%, 184.03%, 148.76%, 93.73%, 87.70%, 153.28%, 64.01%, 172.59%, 56.01%, and 223.53%.

Dietary Fiber: According to the U.S. Food and Drug Administration (FDA), dietary fiber encompasses non-digestible soluble and insoluble carbohydrates (with a degree of polymerization of three or more monomeric units) and lignin, which confer physiological health benefits [67]. Dietary fiber comprises the indigestible remnants of edible plant components, including polysaccharides (e.g., cellulose, hemicellulose, pectins), lignin, gums, mucilage, and oligosaccharides, which resist enzymatic digestion in the human gastrointestinal tract [68].

Dietary fiber contributes to gastrointestinal health by promoting intestinal motility, preventing constipation, and regulating blood glucose and lipid levels [49]. In the current study, dietary fiber content at the R6 stage ranged from 5.06 to 5.78 g/100 g (fresh weight basis). Upon maturation to the R8 stage, dietary fiber content significantly increased across all genotypes, with values ranging from 18.48 to 22.63 g/100 g. Compared to the R6 stage, AVSB2001, AVSB2002, AVSB2004, AVSB2006, AVSB2007, AVSB2009, AVSB2012, AVSB2013, Swarna Vasundhara, Karune, AGS447, and DSb34 showed increases of 251.89%, 270.48%, 227.66%, 238.23%, 240.73%, 238.39%, 265.31%, 255.47%, 289.60%, 261.55%, 277.43%, and 347.23%, respectively. These results are consistent with earlier findings by Faradilla [69].

Total Sugars: Total sugars comprise all free monosaccharides and disaccharides, including glucose, fructose, and sucrose [62]. Among these, sucrose is the predominant sugar contributing to the characteristic sweetness of vegetable soybeans. There was considerable genotypic variability in sucrose and total sugar content. At the R6 stage, total sugar content varied from 2.80 g/100 g (DSb34) to 3.83 g/100 g (Karune). All vegetable soybean genotypes, except AVSB2013, exhibited higher total sugar content than the grain-type control (DSb34). However, at the R8 stage, a consistent reduction in total sugar levels was observed, with concentrations ranging from 1.97 g/100 g (DSb34) to 2.54 g/100 g (AGS447). Despite this decline, the total sugar content of all vegetable soybean genotypes remained significantly higher than that of the grain-type control at the R8 stage. The reduction in total sugar content from the R6 to the R8 stage was notable across genotypes. The percent decrease in AVSB2001, AVSB2002, AVSB2004, AVSB2006, AVSB2007, AVSB2009, AVSB2012, AVSB2013, Swarna Vasundhara, Karune, AGS447, and DSb34 was 32.88%, 32.24%, 24.55%, 22.52%, 23.13%, 20.56%, 21.83%, 17.51%, 26.75%, 39.16%, 29.64%, and 17.26%, respectively.

Reducing and Non-Reducing Sugars: Reducing sugars, including glucose and fructose, were quantified at both developmental stages. At R6, reducing sugar content ranged from 1.43 g/100 g (DSb34) to 2.09 g/100 g (AVSB2007). At R8, concentrations declined, ranging between 0.86 and 0.97 g/100 g. Nonetheless, reducing sugar content in all vegetable soybean genotypes remained significantly higher than in the grain-type control at both stages. Non-reducing sugars, primarily sucrose, were also assessed. At the R6 stage, non-reducing sugar content varied from 0.97 g/100 g (AVSB2007) to 1.81 g/100 g (Karune). At R8, values ranged between 1.03 g/100 g (AVSB2001) and 1.51 g/100 g (DSb34). Interestingly, genotypes such as AVSB2004, AVSB2007, AVSB2009, AVSB2012, AVSB2013, and DSb34 showed an increase in non-reducing sugar content at R8 compared to R6, potentially due to hydrolytic changes and enzymatic modifications during seed maturation (Table 3).

The analysis highlights significant developmental and genotypic variations in vegetable soybeans’ available carbohydrates, dietary fiber, and sugar composition. Notably, maturation from the R6 to the R8 stage is associated with a substantial increase in dietary fiber and available carbohydrates. At the same time, total and reducing sugars tend to decrease, reflecting biochemical transformations during seed development (Table 3 and Table 4). These nutritional profiles are crucial for selecting genotypes for functional food applications and enhancing soybean-based diets’ glycemic and dietary fiber quality.

In Vitro Protein Digestibility of Vegetable Soybean Genotypes and Its Limitations: One of the principal limitations in the application of soybean proteins for the development of functional food products and meat analogues is their association with allergenic potential and the presence of antinutritional factors. These antinutritional compounds can impair protein digestibility and increase nitrogen excretion in feces, thereby reducing the nutritional efficiency of the ingested protein.

**Figure 4:**
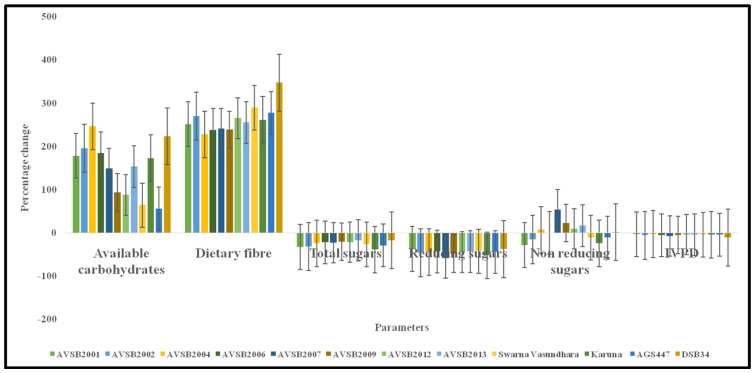
Percentage change in sugars and in vitro protein digestibility of selected vegetable soybean genotypes when compared to the R6 stage.

Protein digestibility refers to how dietary protein is broken down into absorbable amino acids by proteolytic enzymes in the gastrointestinal tract [70]. It is a crucial determinant of protein quality because the physiological utility of dietary amino acids depends not only on their presence in the food matrix but also on their bioavailability following digestion [71,72]. Several factors influence protein digestibility in legumes, particularly the protein’s physicochemical properties, such as quantity, structural conformation, kinetic stability, and inhibitory components within the seed matrix. Key antinutritional compounds that negatively impact digestibility include trypsin inhibitors, phytic acid, tannins, and lectins, all of which interfere with enzymatic hydrolysis of proteins during digestion [73]. To assess the effect of developmental stage on protein digestibility, in vitro protein digestibility (IVPD) of selected vegetable soybean genotypes was evaluated at both the R6 (immature) and R8 (mature) stages (Figure 4).

At the R6 stage, IVPD values ranged from 78.99% to 86.93%, with the highest digestibility observed in the genotype Karune and the lowest in AVSB2009. All genotypes, including the grain-type control (DSb34), exhibited good digestibility, indicating suitability for early-stage harvest in food formulations. In contrast, a general decline in IVPD was observed at the R8 stage, with values ranging from 77.07% (AVSB2004) to 81.87% (AGS447). This reduction in digestibility at the mature stage is consistent with the findings of Calvez et al. [50], who reported a 70–90% range for in vitro protein digestibility in soybeans. The relative decline in IVPD from the R6 to R8 stage among genotypes was as follows: AVSB2001 (−3.66%), AVSB2002 (−6.00%), AVSB2004 (−2.86%), AVSB2006 (−5.88%), AVSB2007 (−7.60%), AVSB2009 (−5.10%), AVSB2012 (−4.02%), AVSB2013 (−4.42%), Swarna Vasundhara (−4.75%), Karune (−4.47%), AGS447 (−4.89%), and DSb34 (−11.24%).

Sripathy and Groot [74] and Weber et al. [75] explained that seed development proceeds through a pre-storage phase—marked by maternal control, cell division, and morphogenesis—and a maturation phase, during which the embryo stops proliferating, undergoes cell expansion, accumulates storage reserves, and functions as a specialized storage organ. Physiological maturity is reached at the culmination of dry matter accumulation and peak differentiation, immediately before the onset of desiccation. This reduction in digestibility at the R8 stage with seed maturity may be attributed to increased deposition of storage proteins with complex quaternary structures and a concomitant rise in antinutritional factors, which may inhibit proteolytic enzyme access or activity. These findings emphasize the importance of harvesting stage selection in maximizing protein bioavailability, particularly when developing vegetable soybean-based food products for human nutrition.

**Table 3:** In vitro protein digestibility, total, reducing, and non-reducing sugar content of R6-stage vegetable soybean (g/100 g).

Genotypes	Available Carbohydrates	Dietary Fiber	Toal Sugars	Reducing Sugars	Non-Reducing Sugars	IVPD (%)
AVSB2001	5.55 ± 0.58 ^cd^	5.34 ± 0.05 ^b^	2.95 ± 0.06 ^cd^	1.52 ± 0.01 ^ab^	1.44 ± 0.05 ^d^	80.94 ± 0.36 ^bcd^
AVSB2002	6.35 ± 0.11 ^de^	5.25 ± 0.01 ^ab^	3.04 ± 0.05 ^cd^	1.64 ± 0.01 ^cde^	1.40 ± 0.03 ^d^	81.77 ± 0.25 ^de^
AVSB2004	5.13 ± 0.28 ^bc^	5.64 ± 0.07 ^c^	2.81 ± 0.01 ^a^	1.66 ± 0.00 ^de^	1.14 ± 0.01 ^bc^	79.34 ± 0.46 ^a^
AVSB2006	4.57 ± 0.44 ^b^	5.78 ± 0.00 ^c^	2.93 ± 0.03 ^bc^	1.52 ± 0.03 ^ab^	1.41 ± 0.04 ^d^	82.05 ± 0.39 ^e^
AVSB2007	4.45 ± 0.06 ^b^	5.72 ± 0.02 ^c^	3.07 ± 0.01 ^d^	2.09 ± 0.01 ^f^	0.97 ± 0.02 ^a^	80.46 ± 0.26 ^b^
AVSB2009	6.06 ± 0.39 ^de^	5.73 ± 0.04 ^c^	2.82 ± 005 ^a^	1.71 ± 0.07 ^e^	1.11 ± 0.03 ^bbc^	78.99 ± 0.16 ^a^
AVSB2012	6.75 ± 0.47 ^e^	5.65 ± 0.03 ^c^	2.84 ± 0.01 ^ab^	1.65 ± 0.00 ^b^	1.19 ± 0.02 ^bc^	81.41 ± 0.24 ^cde^
AVSB2013	4.88 ± 0.37 ^bc^	5.66 ± 0.02 ^c^	2.74 ± 0.07 ^a^	1.54 ± 0.10 ^cde^	1.19 ± 0.04 ^bc^	80.66 ± 0.23 ^bc^
Swarna Vasundhara	4.64 ± 0.11 ^b^	5.29 ± 0.26 ^b^	3.29 ± 0.01 ^e^	1.59 ± 0.00 ^bcd^	1.69 ± 0.00 ^e^	83.50 ± 0.46 ^f^
Karune	4.89 ± 0.57 ^bc^	5.28 ± 0.01 ^b^	3.83 ± 0.01 ^f^	2.02 ± 0.01 ^f^	1.81 ± 0.02 ^f^	84.64 ± 0.48 ^g^
AGS447	5.66 ± 0.13 ^cd^	5.76 ± 0.01 ^c^	3.07 ± 0.07 ^d^	1.56 ± 0.01 ^bc^	1.24 ± 0.07 ^c^	86.08 ± 0.31 ^h^
DSb34	3.40 ± 0.04 ^a^	5.06 ± 0.07 ^a^	2.80 ± 0.06 ^a^	1.43 ± 0.01 ^a^	1.63 ± 0.07 ^e^	86.93 ± 0.56 ^h^
F value	20.55	24.92	80.51	130.11	111.11	142.14
*p* value	0.00 **	0.00 **	0.00 **	0.00 **	0.00 **	0.00 **

**Note:** The values are presented as the mean ± SD of (n = 3) replications. ** Significant at 1%. Values with a different superscript in the same column are significantly different (*p* ≤ 0.05).

**Table 4:** In vitro protein digestibility, total, reducing, and non-reducing sugar content of R8-stage vegetable soybean genotypes (g/100 g).

Genotypes	Available Carbohydrates	Dietary Fiber	Toal Sugars	Reducing Sugars	Non-Reducing Sugars	IVPD (%)
AVSB2001	15.44 ± 1.44 ^de^	18.77 ± 0.83 ^ab^	1.98 ± 0.03 ^a^	0.95 ± 0.01 ^ef^	1.03 ± 0.01 ^a^	77.97 ± 0.37 ^d^
AVSB2002	18.80 ± 0.41 ^f^	19.45 ± 0.88 ^ab^	2.06 ± 0.01 ^ab^	0.88 ± 0.02 ^ab^	1.18 ± 0.01 ^b^	76.86 ± 0.14 ^c^
AVSB2004	17.78 ± 0.78 ^ef^	18.48 ± 0.07 ^a^	2.12 ± 0.03 ^b^	0.92 ± 0.01 ^cde^	1.21 ± 0.03 ^bc^	77.07 ± 0.10 ^c^
AVSB2006	12.98 ± 0.39 ^cd^	19.55 ± 0.41 ^ab^	2.27 ± 0.01 ^cd^	0.86 ± 0.02 ^a^	1.31 ± 0.03 ^de^	77.22 ± 0.13 ^c^
AVSB2007	11.07 ± 1.70 ^bc^	19.49 ± 0.47 ^ab^	2.36 ± 0.05 ^de^	0.87 ± 0.00 ^a^	1.49 ± 0.05 ^e^	74.34 ± 0.24 ^a^
AVSB2009	11.74 ± 2.03 ^bc^	19.39 ± 1.11 ^ab^	2.24 ± 0.04 ^c^	0.88 ± 0.00 ^abc^	1.36 ± 0.01 ^d^	74.96 ± 0.11 ^b^
AVSB2012	12.67 ± 1.15 ^cd^	20.64 ± 0.19 ^bc^	2.22 ± 0.09 ^c^	0.92 ± 0.03 ^de^	1.30 ± 0.08 ^cd^	78.13 ± 0.19 ^d^
AVSB2013	12.36 ± 0.39 ^cd^	20.12 ± 0.54 ^abc^	2.26 ± 0.01 ^cd^	0.87 ± 0.02 ^ab^	1.39 ± 0.02 ^de^	77.09 ± 0.09 ^c^
Swarna Vasundhara	7.61 ± 0.83 ^a^	20.61 ± 1.63 ^bc^	2.41 ± 0.11 ^e^	0.91 ± 0.01 ^bcd^	1.50 ± 0.04 ^e^	79.53 ± 0.22 ^e^
Karune	13.33 ± 33 ^cd^	19.09 ± 0.93 ^ab^	2.33 ± 0.05 ^cde^	0.97 ± 0.02 ^f^	1.36 ± 0.11 ^d^	80.85 ± 0.15 ^e^
AGS447	8.83 ± 1.78 ^ab^	21.74 ± 0.65 ^cd^	2.54 ± 0.04 ^f^	0.87 ± 0.01 ^ab^	1.10 ± 0.01 ^ab^	81.87 ± 0.44 ^f^
DSb34	11.00 ± 0.82 ^bc^	22.63 ± 0.67 ^d^	1.97 ± 0.01 ^a^	0.89 ± 0.00 ^abcd^	1.51 ± 0.02 ^e^	77.16 ± 0.14 ^c^
F value	20.86	6.90	51.75	16.15	43.94	249.87
*p* value	0.00 **	0.00 **	0.00 **	0.00 **	0.00 **	0.00 **

**Note:** The values are presented as the mean ± SD of (n = 3) replications. ** Significant at 1%. Values with a different superscript in the same column are significantly different (*p* ≤ 0.05).

#### 3.2.3. Mineral Composition of Vegetable Soybean Genotypes at the R6 and R8 Stage

Minerals are essential nutrients that are critical in maintaining overall physical and mental well-being. They are integral to the structure and function of bones, teeth, blood, muscles, tissues, and nerve cells. They are involved in acid–base balance, nerve response to physiological stimuli, and blood coagulation [76]. The concentrations (mg/100 g fresh weight basis) of key minerals—calcium, magnesium, iron, zinc, sodium, manganese, phosphorus, potassium, and copper—across vegetable soybean genotypes at the R6 (green pod) and R8 (mature seed) developmental stages are presented in Table 5 and Table 6.

Calcium: At the R6 stage, calcium content ranged from 112.43 mg/100 g (AVSB2012) to 140.78 mg/100 g (AVSB2001). In the R8 stage, calcium content increased substantially, ranging from 243.49 mg/100 g (Swarna Vasundhara) to 356.11 mg/100 g (AVSB2001). The relative increase in calcium content from R6 to R8 ranged between 94.76% and 1668.13% across genotypes. Calcium is a critical micromineral necessary for bone and teeth formation, blood clotting, neurotransmission, muscle contraction, hormonal function, and maintenance of normal cardiac rhythm [77]. The increase in mineral content at the R8 stage might be because of the accumulation of ash content during seed maturation.

Iron: Iron is vital for haemoglobin formation and oxygen transport and is essential in preventing iron deficiency anaemia [78,79]. The FAO/WHO recommends a daily iron intake of 13.7 mg to avoid anaemia and related disorders [17,80]. At R6, iron content ranged from 4.33 mg/100 g (AGS447) to 4.63 mg/100 g (Karune). At R8, iron content increased significantly, from 9.46 mg/100 g (Swarna Vasundhara) to 12.19 mg/100 g (AGS447). The percentage increase in iron content across genotypes ranged from 153.35% to 287.83%. A 100 g serving of these soybeans contributes approximately 20.36–33.79% of the recommended daily iron intake.

Copper: Copper plays a role in vascular, neurological, and immune function and facilitates iron absorption [81]. No significant differences were observed in copper levels at the R6 stage, but values at the R8 stage ranged from 0.19 to 0.39 mg/100 g. Increases from R6 to R8 were modest, with the highest increase observed in AVSB2012 and AVSB2013 (69.56%).

Zinc: Zinc is crucial for immune function, wound healing, cell division, and sensory perception [17,76,82]. The RDA for zinc is 17.0 mg/day for men and 13.2 mg/day for women [83]. At R6, zinc content ranged from 1.13 mg/100 g (AVSB2004) to 1.76 mg/100 g (AVSB2012). At R8, values increased substantially, from 2.89 mg/100 g (DSb34) to 3.96 mg/100 g (AVSB2002). Percent increases from R6 to R8 ranged from 74.09% to 199.12%.

Magnesium: Magnesium supports bone health and immune function, and its intake has been linked to enhanced T-cell activity and protection against infections and cancers [17]. At R6, magnesium content ranged from 61.29 mg/100 g (AVSB2009) to 77.51 mg/100 g (Karune). At R8, values increased markedly, ranging from 192.92 mg/100 g (Swarna Vasundhara) to 261.04 mg/100 g (AGS447). Percent increases ranged from 180.65% to 260.90%.

Manganese: At R6, no significant variation was observed in manganese levels. At R8, manganese content ranged from 2.14 mg/100 g (AVSB2009) to 4.32 mg/100 g (AGS447). Increases from R6 to R8 ranged from 31.28% to 355.40%, with the highest increase observed in DSb34.

Potassium: Potassium acts as a vasodilator and is crucial in reducing blood pressure and maintaining overall cellular function [79]. At R6, potassium content ranged from 443.07 mg/100 g (AVSB2002) to 576.33 mg/100 g (AVSB2004). At R8, values increased significantly, with Karune showing the highest content (1700.00 mg/100 g), followed by AVSB2001 (1608.17 mg/100 g). Percent increases ranged from 139.45% to 232.16%.

Sodium: Sodium is essential for maintaining blood pressure and neuromuscular function [84]. At R6, sodium content ranged from 1.11 mg/100 g (AVSB2001) to 3.86 mg/100 g (AGS447). At R8, values ranged from 4.73 mg/100 g (AVSB2013) to 8.12 mg/100 g (AVSB2004), with percent increases from 76.68% to 342.34%.

**Figure 5:**
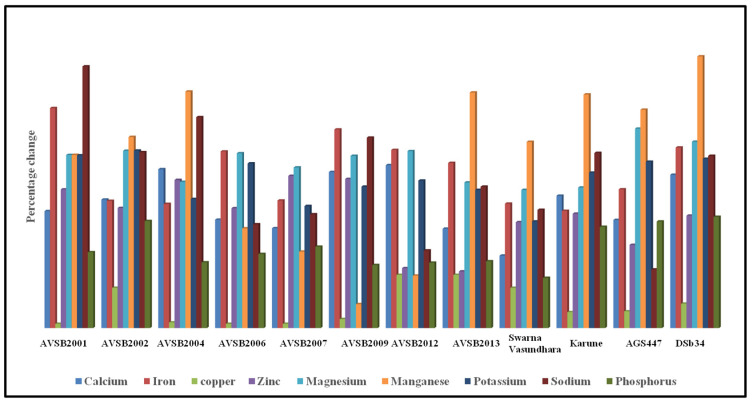
Percentage change in mineral composition of vegetable soybean genotypes from the R6 to R8 stage.

Phosphorus: Phosphorus is closely associated with calcium in bone mineralization and structural integrity [49]. At R6, phosphorus content ranged from 51.12 mg/100 g (AVSB2002) to 72.19 mg/100 g (AVSB2013). At R8, phosphorus content ranged from 106.65 mg/100 g (Swarna Vasundhara) to 148.09 mg/100 g (Karune). Percent increases across genotypes ranged from 65.73% to 145.52% (Figure 5). Overall, the mineral profile of vegetable soybean genotypes significantly improved from the R6 to the R8 stage. These findings reinforce the nutritional value of mature vegetable soybeans and their potential contribution to dietary mineral intake.

**Table 5:** Mineral composition of vegetable soybean genotypes at the R6 stage (mg/100 g).

Genotypes	Calcium	Iron	Copper	Zinc	Magnesium	Manganese	Potassium	Sodium	Phosphorus
AVSB2001	140.78 ± 0.97 ^f^	3.04 ± 0.35 ^ab^	0.18 ± 0.00 ^a^	1.29 ± 0.13 ^a^	65.81 ± 0.82 ^abc^	0.98 ± 0.17 ^ab^	493.43 ± 0.34 ^abc^	1.11 ± 0.04 ^a^	54.83 ± 0.02 ^ab^
AVSB2002	131.23 ± 0.14 ^ef^	4.35 ± 0.22 ^ab^	0.19 ± 0.00 ^a^	1.54 ± 0.01 ^a^	69.78 ± 0.35 ^cd^	1.02 ± 0.14 ^ab^	443.07 ± 0.25 ^a^	2.19 ± 0.23 ^abc^	51.12 ± 0.61 ^a^
AVSB2004	114.89 ± 0.53 ^ab^	3.99 ± 1.53 ^ab^	0.27 ± 0.17 ^a^	1.13 ± 0.77 ^a^	70.22 ± 2.89 ^cd^	0.74 ± 0.12 ^a^	576.33 ± 1.87 ^e^	2.16 ± 0.13 ^abc^	64.38 ± 0.26 ^d^
AVSB2006	132.52 ± 1.07 ^ef^	3.22 ± 0.15 ^ab^	0.22 ± 0.04 ^a^	1.41 ± 0.15 ^a^	63.49 ± 1.62 ^ab^	1.02 ± 0.15 ^ab^	457.26 ± 1.37 ^ab^	3.30 ± 0.50 ^cd^	58.55 ± 0.18 ^bc^
AVSB2007	134.50 ± 0.78 ^ef^	3.98 ± 0.74 ^ab^	0.18 ± 0.00 ^a^	1.14 ± 0.22 ^a^	65.91 ± 0.06 ^abc^	1.12 ± 0.18 ^ab^	552.81 ± 1.21 ^de^	2.19 ± 1.24 ^abc^	65.23 ± 0.38 ^d^
AVSB2009	116.52 ± 2.28 ^abc^	2.79 ± 0.14 ^a^	0.34 ± 0.08 ^a^	1.22 ± 0.06 ^a^	61.29 ± 0.33 ^a^	1.63 ± 0.86 ^b^	503.63 ± 0.50 ^bcd^	1.87 ± 0.70 ^abc^	66.57 ± 0.05 ^d^
AVSB2012	111.43 ± 1.51 ^a^	3.48 ± 0.24 ^ab^	0.23 ± 0.05 ^a^	1.76 ± 0.00 ^a^	67.17 ± 0.33 ^bcd^	1.34 ± 0.59 ^ab^	533.13 ± 1.13 ^cde^	2.53 ± 0.16 ^abcd^	58.40 ± 0.20 ^bc^
AVSB2013	127.49 ± 0.29 ^cde^	3.18 ± 0.04 ^ab^	0.23 ± 0.05 ^a^	1.66 ± 0.05 ^a^	68.04 ± 0.71 ^bcd^	0.85 ± 0.09 ^ab^	563.97 ± 0.31 ^e^	1.66 ± 0.05 ^ab^	72.19 ± 0.40 ^e^
Swarna Vasundhara	125.02 ± 0.07 ^bcde^	3.60 ± 0.28 ^ab^	0.19 ± 0.00 ^a^	1.35 ± 0.03 ^a^	68.74 ± 1.38 ^bcd^	0.94 ± 0.11 ^ab^	566.68 ± 1.34 ^e^	2.80 ± 0.33 ^bcd^	64.35 ± 0.85 ^d^
Karune	123.12 ± 0.84 ^def^	4.63 ± 0.77 ^b^	0.24 ± 0.04 ^a^	1.37 ± 0.04 ^a^	77.51 ± 2.26 ^e^	0.89 ± 0.14 ^ab^	560.42 ± 1.24 ^e^	1.99 ± 0.66 ^abc^	63.71 ± 0.61 ^cd^
AGS447	119.96 ± 0.25 ^abcd^	4.33 ± 0.74 ^ab^	0.32 ± 0.05 ^a^	1.47 ± 0.13 ^a^	72.33 ± 0.25 ^d^	1.12 ± 0.00 ^ab^	492.35 ± 0.38 ^abc^	3.86 ± 0.05 ^d^	54.33 ± 0.66 ^ab^
DSb34	118.41 ± 0.94 ^abc^	3.07 ± 0.37 ^ab^	0.22 ± 0.05 ^a^	1.21 ± 0.09 ^a^	66.48 ± 1.00 ^abc^	0.74 ± 0.00 ^a^	491.30 ± 1.81 ^abc^	2.05 ± 1.22 ^abc^	52.96 ± 3.02 ^ab^
F value	10.97	2.89	2.08	1.96	10.78	1.75	12.63	4.34	24.37
*p* value	0.00 **	0.01 *	0.06 ^NS^	0.08 ^NS^	0.00 **	0.12 ^NS^	0.00 **	0.00 **	0.00 **

**Note:** The values are presented as the mean ± SD of (n = 3) replications. ^NS^—non-significant, * Significant at 5%, ** Significant at 1%. Values with a different superscript in the same column are significantly different (*p* ≤ 0.05).

**Table 6:** Mineral composition of vegetable soybean genotypes at the R8 stage (mg/100 g).

Genotypes	Calcium	Iron	Copper	Zinc	Magnesium	Manganese	Potassium	Sodium	Phosphorus
AVSB2001	356.11 ± 0.12 ^f^	11.79 ± 0.00 ^e^	0.19 ± 0.00 ^a^	3.63 ± 0.09 ^cd^	214.87 ± 1.31 ^de^	3.20 ± 0.14 ^bc^	1608.17 ± 1.05 ^c^	4.91 ± 0.39 ^a^	109.26 ± 0.43 ^a^
AVSB2002	351.87 ± 1.39 ^f^	11.59 ± 0.03 ^e^	0.29 ± 0.10 ^ab^	3.96 ± 0.37 ^d^	231.64 ± 4.17 ^h^	3.57 ± 0.18 ^bc^	1471.69 ± 0.27 ^b^	7.23 ± 0.25 ^bc^	122.73 ± 0.59 ^c^
AVSB2004	353.67 ± 0.38 ^f^	10.47 ± 0.07 ^cd^	0.29 ± 0.09 ^ab^	3.32 ± 0.07 ^bc^	204.52 ± 0.19 ^bc^	3.03 ± 0.23 ^b^	1549.23 ± 0.28 ^c^	8.12 ± 0.27 ^c^	119.74 ± 0.24 ^c^
AVSB2006	320.41 ± 0.48 ^d^	10.66 ± 0.13 ^d^	0.19 ± 0.00 ^a^	3.62 ± 0.08 ^cd^	208.71 ± 0.36 ^cd^	2.35 ± 0.00 ^a^	1442.45 ± 1.12 ^b^	7.78 ± 0.85 ^cd^	115.29 ± 0.42 ^b^
AVSB2007	310.24 ± 1.03 ^c^	10.62 ± 0.02 ^d^	0.19 ± 0.00 ^a^	3.41 ± 0.12 ^bc^	204.36 ± 4.56 ^bc^	2.24 ± 0.11 ^a^	1435.87 ± 0.27 ^b^	5.45 ± 0.35 ^a^	134.67 ± 4.17 ^e^
AVSB2009	354.47 ± 0.34 ^f^	10.04 ± 0.12 ^b^	0.19 ± 0.00 ^a^	3.60 ± 0.08 ^cd^	199.42 ± 0.74 ^ab^	2.14 ± 0.00 ^a^	1435.37 ± 0.85 ^b^	6.53 ± 0.11 ^b^	121.47 ± 0.60 ^c^
AVSB2012	348.93 ± 0.67 ^f^	11.59 ± 0.12 ^e^	0.39 ± 0.00 ^b^	3.14 ± 0.18 ^ab^	222.68 ± 0.56 ^fg^	2.26 ± 0.08 ^a^	1561.72 ± 1.76 ^c^	5.10 ± 0.36 ^a^	108.34 ± 2.05 ^a^
AVSB2013	293.28 ± 1.58 ^b^	10.05 ± 0.05 ^b^	0.39 ± 0.00 ^b^	2.89 ± 0.01 ^a^	197.64 ± 0.86 ^ab^	3.47 ± 0.37 ^bc^	1583.32 ± 0.69 ^c^	4.73 ± 0.12 ^a^	135.27 ± 1.14 ^e^
Swarna Vasundhara	243.49 ± 1.40 ^a^	9.46 ± 0.17 ^a^	0.29 ± 0.09 ^ab^	3.22 ± 0.33 ^abc^	192.92 ± 0.24 ^a^	3.23 ± 0.53 ^bc^	1356.94 ± 0.16 ^a^	7.13 ± 0.38 ^bc^	106.65 ± 0.86 ^a^
Karune	336.25 ± 0.20 ^e^	11.73 ± 0.04 ^e^	0.29 ± 0.09 ^ab^	3.42 ± 0.08 ^bc^	219.99 ± 1.07 ^ef^	3.61 ± 0.08 ^c^	1700.00 ± 0.91 ^d^	6.55 ± 0.07 ^b^	148.09 ± 0.66 ^f^
AGS447	289.66 ± 0.50 ^b^	12.19 ± 0.12 ^f^	0.39 ± 0.00 ^b^	3.07 ± 0.01 ^ab^	261.04 ± 0.53 ^i^	4.32 ± 0.08 ^d^	1563.67 ± 0.68 ^c^	6.82 ± 0.07 ^b^	130.05 ± 0.21 ^d^
DSb34	355.89 ± 0.55 ^f^	10.32 ± 0.11 ^c^	0.29 ± 0.00 ^ab^	2.99 ± 0.09 ^ab^	228.58 ± 0.51 ^g^	3.37 ± 0.09 ^bc^	1579.41 ± 0.45 ^c^	6.67 ± 0.29 ^b^	130.03 ± 0.23 ^d^
F value	435.55	262.19	6.36	10.20	128.35	29.92	110.86	29.36	159.89
*p* value	0.00 **	0.00 **	0.00 **	0.00 **	0.00 **	0.00 **	0.00 **	0.00 **	0.00 **

**Note:** The values are presented as the mean ± SD of (n = 3) replications. ** Significant at 1%. Values with a different superscript in the same column are significantly different (*p* ≤ 0.05).

#### 3.2.4. Mineral: Mineral Ratios of Selected Vegetable Soybean Genotypes at the R6 and R8 Stage

Mineral-to-mineral ratios are crucial indicators of nutritional quality, as interactions between minerals can significantly influence their bioavailability and physiological efficacy in the human body. Some minerals enhance while others inhibit specific nutrient absorption or metabolic utilization [85]. In this context, key mineral ratios were calculated for twelve vegetable soybean genotypes at both the R6 (green pod) and R8 (physiological maturity) stages, and the results are summarized in Table 7.

Sodium-to-Potassium Ratio (Na:K): The sodium-to-potassium (Na:K) ratio is a critical dietary index for cardiovascular health, particularly in managing and preventing hypertension and stroke. According to the World Health Organization (WHO, 2006), a dietary Na:K ratio below 1.0 is recommended to control high blood pressure effectively [80]. At the R6 stage, Na:K ratios ranged from 0.0022 (AVSB2001) to 0.0078 (AVSB2006), and at the R8 stage, the values varied between 0.0029 (AVSB2013) and 0.0054 (AVSB2006). All tested genotypes exhibited Na:K ratios far below the recommended upper limit of 1.0 at both stages. This suggests that both immature and mature vegetable soybeans are potentially beneficial for hypertensive individuals and can contribute to lowering blood pressure.

Calcium-to-Phosphorus Ratio (Ca:P): The calcium-to-phosphorus (Ca:P) ratio is key to skeletal health and mineral utilisation efficiency. A ratio greater than 0.5 is essential for optimal bone development, while values near or above 1.0 enhance calcium and phosphorus bioavailability [86]. At the R6 stage, the Ca:P ratios ranged from 1.7503 (AVSB2009) to 2.5675 (AVSB2001). At R8, values increased slightly, ranging between 2.1681 (AVSB2013) and 3.2592 (AVSB2001). All genotypes at both developmental stages exceeded the critical threshold of 0.5, indicating that vegetable soybeans are highly favourable for supporting bone mineralisation and preventing calcium-phosphorus imbalances.

Calcium-to-Potassium Ratio (Ca:K): The calcium-to-potassium (Ca:K) ratio, often referred to as the “thyroid ratio”, is associated with endocrine regulation, particularly thyroid function. A low Ca:K ratio indicates enhanced thyroid activity, which may be beneficial under certain metabolic conditions [86]. At R6, Ca:K ratios ranged from 0.1993 (AVSB2004) to 0.2962 (AVSB2002). R8 values were slightly lower, ranging between 0.1794 (Swarna Vasundhara) and 0.2470 (AVSB2009). All genotypes showed Ca:K ratios below standard reference values, suggesting a stimulatory effect on thyroid function. Thus, vegetable soybeans may support optimal thyroid activity, particularly in individuals with subclinical hypothyroidism or sluggish metabolism.

Iron-to-Zinc Ratio (Fe:Zn): Iron and zinc exhibit competitive interactions in intestinal absorption pathways, particularly at elevated intake levels. Studies have shown that an Fe:Zn molar ratio below 2:1 is optimal, while ratios up to 5:1–10:1 show a dose-dependent but tolerable reduction in zinc absorption [87]. At R6, Fe:Zn ratios ranged from 1.9157 (AVSB2013) to 3.5310 (AVSB2004). At R8, the ratios varied between 2.7880 and 3.9706. All values remained within the physiologically acceptable range, implying that iron levels in these genotypes are unlikely to impair zinc uptake significantly, and both micronutrients can be co-utilised effectively from these soy-based foods.

**Table 7:** Mineral–mineral ratios of vegetable soybean genotypes at R6 and R8 stages.

	R6 Stage				R8 Stage			
Genotypes	Na:K	Ca:P	Ca:K	Fe:Zn	Na:K	Ca:P	Ca:K	Fe:Zn
AVSB2001	0.0022	2.5676	0.2853	2.3566	0.0031	3.2593	0.2214	3.2479
AVSB2002	0.0049	2.5671	0.2962	2.8247	0.0049	2.8670	0.2391	2.9268
AVSB2004	0.0037	1.7846	0.1993	3.5310	0.0052	2.9536	0.2283	3.1536
AVSB2006	0.0072	2.2634	0.2898	2.2837	0.0054	2.7792	0.2221	2.9448
AVSB2007	0.0040	2.0619	0.2433	3.4912	0.0038	2.3037	0.2161	3.1144
AVSB2009	0.0037	1.7503	0.2314	2.2869	0.0045	2.9182	0.2470	2.7889
AVSB2012	0.0047	1.9080	0.2090	1.9773	0.0033	3.2207	0.2234	3.6911
AVSB2013	0.0029	1.7660	0.2261	1.9157	0.0030	2.1681	0.1852	3.4775
Swarna Vasundhara	0.0049	1.9428	0.2206	2.6667	0.0053	2.2831	0.1794	2.9379
Karune	0.0036	1.9325	0.2197	3.3796	0.0039	2.2706	0.1978	3.4298
AGS447	0.0078	2.2080	0.2436	2.9456	0.0044	2.2273	0.1852	3.9707
DSb34	0.0042	2.2358	0.2410	2.5372	0.0042	2.7370	0.2253	3.4515
WHO, 2006	<1	>0.5	<4	>2	<1	>0.5	<4	>2

A comprehensive evaluation was conducted to identify the most suitable vegetable soybean genotypes for consumption and further development, considering sensory attributes, proximate composition, sugar content, in vitro protein digestibility, and mineral profile at both R6 (green pod) and R8 (physiological maturity) stages. Based on the integrated assessment of these parameters, the genotypes Swarna Vasundhara, Karune, and AGS447 emerged as the best performers at the R6 stage, demonstrating superior nutritional qualities and sensory acceptability. At the R8 stage, AVSB2001, AVSB2004, and AGS447 were identified as promising candidates for further study. Although AGS447 exhibited lower sensory acceptance at the R8 stage when boiled, it showed favorable nutritional characteristics. Its acceptability can be improved through alternative processing methods such as roasting, extrusion, or germination, which may enhance its sensory appeal while preserving its nutritional value.

### 3.3. Percentage Adequacy of Recommended Dietary Allowances (RDA) from the Consumption of Selected Vegetable Soybean Genotypes at R6 and R8 Stages

Adequate nutrition is fundamental for maintaining health, supporting growth, and ensuring productive life. While RDAs provide nutrient-specific targets, current nutrition science emphasizes a food-based approach to meet dietary needs effectively [88]. Vegetable soybean, a short-duration legume crop, is a rich source of high-quality protein, essential minerals, vitamins, and bioactive compounds. Given its nutrient density, this study estimated the percentage adequacy of RDA met by consuming selected vegetable soybean genotypes (fresh vegetables or snacks) for different age and occupational groups. The findings are presented in Table 8, Table 9, Table 10 and Table 11.

#### 3.3.1. Percentage Adequacy of RDA from R6- and R8-Stage Vegetable Soybean Genotypes in Adults

The Indian Council of Medical Research—National Institute of Nutrition (ICMR-NIN, 2024 [83]) has recommended dietary intakes for adult men with varying physical activity levels as follows: energy requirements range from 2110 kcal (sedentary) to 3470 kcal (heavy work), with protein at 54 g, fat between 25 to 40 g, dietary fiber at 30 g, carbohydrates at 100 g, calcium at 1000 mg, phosphorus at 600 mg, magnesium at 440 mg, potassium at 3500 mg, iron at 19 mg, and zinc at 17 mg.

The study evaluated the nutritional adequacy of a 100 g serving of selected R6-stage genotypes for men. For instance, Swarna Vasundhara contributed 4.24–6.97% of energy, 21.15% of protein, 17.13–27.40% of fat, 9.93% of carbohydrates, 10.58–17.63% of dietary fiber, 12.5% of calcium, 18.95% of iron, 7.94% of zinc, 15.62% of magnesium, 16.19% of potassium, 10.73% of phosphorus, and 23.50% of manganese. Similarly, Karune provided slightly higher levels of protein (28.31%) and iron (24.37%) along with comparable amounts of other nutrients, while AGS447 also offered substantial nutritional contributions, particularly protein (23.35%) and manganese (28%) (Table 8).

For adult women (aged 19–39 years, approximately 55 kg), the ICMR-NIN recommendations vary slightly but generally include energy requirements from 1660 to 2720 kcal, depending on activity level, with protein at 46 g, fat between 20 and 30 g, and similar levels for fiber and minerals. Consumption of 100 g of Swarna Vasundhara, Karune, or AGS447 was found to provide between 5.07% and 9.48% of energy, 24.83% to 33.24% of protein, 19.83% to 34.45% of fat, 8.47% to 9.93% of carbohydrates, and notable proportions of calcium, iron, zinc, magnesium, potassium, phosphorus, and manganese. Among these, Karune showed the highest overall percentage adequacy for energy, protein, fat, and essential minerals in both men and women.

At the R8 stage, a smaller serving size of 50 g of selected genotypes (AVSB2001, AVSB2004, and AGS447) was assessed for their nutritional adequacy in adults. All genotypes were confirmed to be excellent sources of essential nutrients. Among them, AGS447 consistently provided the highest levels of energy, protein, fat, dietary fiber, iron, phosphorus, magnesium, and manganese across all activity categories for both men and women. Combining low calorie content with high-quality protein, healthy fats, dietary fiber, and essential minerals makes vegetable soybean genotypes a promising option for health-conscious adults (Table 9).

#### 3.3.2. Percentage Adequacy of RDA from Vegetable Soybean Genotypes at R6 and R8 Stages in Children

The study also assessed the contribution of selected vegetable soybean genotypes to children’s dietary needs across different age groups. According to ICMR-NIN (2024), nutritional requirements vary for boys and girls aged 4 to 18. One serving of 100 g of R6-stage genotypes such as Swarna Vasundhara, Karune, and AGS447 met more than 50% of the protein requirements and substantial proportions of fat (23.8–27.56%), dietary fiber (26.4–28.8%), calcium (21.81–22.39%), iron (32.73–42.09%), and zinc (30.44–32.73%) for children aged 4–6 years. Similarly, children aged 7–18 would meet these genotypes by consuming 20.76% and 66.48% of protein needs, 11.90% to 23.80% of fat, and 13.89% to 28.80% of dietary fiber. Karune was the most effective in meeting the RDA across all growing children’s age groups (Table 10 and Table 11).

At the R8 stage, nutritional adequacy was evaluated for smaller servings of 30 g/day of genotypes AVSB2001, AVSB2004, and AGS447. The nutrient requirements for children aged 4–9 years are uniform for both sexes, while older age groups have sex-specific guidelines. The analysis revealed that this small serving size provides between 5.53% and 10.27% of energy, 19.72% to 67.03% of protein, 18.56% to 26.86% of fat, 14.59% to 32.61% of dietary fiber, 8.28% to 15.80% of calcium, 9.82% to 33.25% of iron, 6.49% to 24.20% of zinc, 16.15% to 62.65% of magnesium, anod 5.46% to 11.15% of phosphorus requirements across various age groups. Notably, AGS447 emerged as an excellent contributor to fulfilling the nutritional requirements of growing children, providing a balanced mix of essential nutrients even at small serving sizes.

**Table 8:** Percentage adequacy of RDA met by the consumption of vegetable soybean genotypes at the R6 stage in the adult population (100 g serving size).

Nutrients	Men Sedentary	Men Moderate	Men Heavy	Women Sedentary	Women Moderate	Women Heavy
Swarna Vasundhara						
Energy	6.97	5.43	4.24	8.86	6.91	5.41
Protein	21.15	21.15	21.15	24.83	24.83	24.83
Fat	27.40	22.83	17.13	34.25	27.40	22.83
Total carbohydrates	9.93	9.93	9.93	9.93	9.93	9.93
Dietary fiber	17.63	13.23	10.58	21.16	17.63	13.23
Calcium	12.50	12.50	12.50	12.50	12.50	12.50
Iron	18.95	18.95	18.95	12.41	12.41	12.41
Zinc	7.94	7.94	7.94	10.23	10.23	10.23
Magnesium	15.62	15.62	15.62	18.58	18.58	18.58
Potassium	16.19	16.19	16.19	16.19	16.19	16.19
Phosphorus	10.73	10.73	10.73	10.73	10.73	10.73
Manganese	23.50	23.50	23.50	23.50	23.50	23.50
Karune						
Energy	7.46	5.81	4.53	9.48	7.39	5.79
Protein	28.31	28.31	28.31	33.24	33.24	33.24
Fat	27.56	22.97	17.22	34.45	27.56	22.97
Total carbohydrates	9.03	9.03	9.03	9.03	9.03	9.03
Dietary fiber	17.60	13.20	10.56	21.12	17.6	13.20
Calcium	12.31	12.31	12.31	12.31	12.31	12.31
Iron	24.37	24.37	24.37	15.96	15.96	15.96
Zinc	8.06	8.06	8.06	10.38	10.38	10.37
Magnesium	17.61	17.61	17.61	20.95	20.95	20.95
Potassium	16.01	16.01	16.01	16.01	16.01	16.01
Phosphorus	10.62	10.62	10.62	10.62	10.62	10.62
Manganese	22.25	22.25	22.25	22.25	22.25	22.25
AGS447						
Energy	6.54	5.09	3.98	8.31	6.48	5.07
Protein	23.35	23.35	23.35	27.41	27.41	27.41
Fat	23.80	19.83	14.88	29.75	23.80	19.83
Total carbohydrates	8.47	8.47	8.47	8.47	8.47	8.47
Dietary fiber	19.20	14.40	11.52	23.04	19.20	14.40
Calcium	12.00	12.00	12.00	12.00	12.00	12.00
Iron	22.79	22.79	22.79	14.93	14.93	14.93
Zinc	8.65	8.65	8.65	11.14	11.14	11.14
Magnesium	16.44	16.44	16.44	19.55	19.55	19.55
Potassium	14.07	14.07	14.07	14.07	14.07	14.07
Phosphorus	9.06	9.06	9.06	9.06	9.06	9.06
Manganese	28.00	28.00	28.00	28.00	28.00	28.00

**Table 9:** Percentage adequacy of RDA met by the consumption of vegetable soybean genotypes at the R8 stage in the adult population (50 g serving size).

Nutrients	Men Sedentary	Men Moderate	Men Heavy	Women Sedentary	Women Moderate	Women Heavy
AVSB2001						
Energy	11.03	8.59	6.71	14.02	10.93	8.56
Protein	29.77	29.77	29.77	34.95	34.95	34.95
Fat	44.44	37.03	27.78	55.55	44.44	37.03
Total carbohydrates	17.11	17.11	17.11	17.11	17.11	17.11
Dietary fiber	31.28	23.46	18.77	37.54	31.28	23.46
Calcium	17.81	17.81	17.81	17.81	17.81	17.81
Iron	31.03	31.03	31.03	20.33	20.33	20.33
Zinc	10.68	10.68	10.68	13.75	13.75	13.75
Magnesium	24.42	24.42	24.42	29.04	29.04	29.04
Potassium	22.97	217.32	22.97	22.97	22.97	22.97
Phosphorus	9.11	1.56	9.11	9.11	9.11	9.11
Manganese	40.00	40.00	40.00	40.00	40.00	40.00
AVSB2004						
Energy	10.92	8.50	6.64	13.88	10.82	8.47
Protein	28.00	28.00	28.00	32.87	32.87	32.87
Fat	43.30	36.08	27.06	54.13	43.30	36.08
Total carbohydrates	18.12	18.12	18.12	18.12	18.12	18.12
Dietary fiber	30.80	23.10	18.48	36.96	30.80	23.10
Calcium	17.68	17.68	17.68	17.68	17.68	17.68
Iron	27.55	27.55	27.55	18.05	18.05	18.05
Zinc	9.76	9.76	9.76	12.58	12.58	12.58
Magnesium	23.24	23.24	23.24	27.64	27.64	27.64
Potassium	22.13	209.36	22.13	22.13	22.13	22.13
Phosphorus	9.98	1.71	9.98	9.98	9.98	9.98
Manganese	37.88	37.88	37.88	37.88	37.88	37.88
AGS447						
Energy	11.06	8.61	6.73	14.06	10.96	8.58
Protein	33.10	33.10	33.10	38.86	38.86	38.86
Fat	44.76	37.30	27.98	55.95	44.76	37.30
Total carbohydrates	16.82	16.82	16.82	16.82	16.82	16.82
Dietary fiber	36.23	27.18	21.74	43.48	36.23	27.18
Calcium	14.48	14.48	14.48	14.48	14.48	14.48
Iron	32.08	32.08	32.08	21.02	21.02	21.02
Zinc	9.03	9.03	9.03	11.63	11.63	11.63
Magnesium	29.66	29.66	29.66	35.28	35.28	35.28
Potassium	22.34	211.31	22.34	22.34	22.34	22.34
Phosphorus	10.84	1.86	10.84	10.84	10.84	10.84
Manganese	54.00	54.00	54.00	54.00	54.00	54.00

**Table 10:** Percentage adequacy of RDA met by the consumption of vegetable soybean genotypes at the R6 stage in different age groups of children (100 g serving size).

Nutrients	4–6 yrs	7–9 yrs	10–12 yr	10–12 yr	13–15 yrs	13–15 yrs	16–18 yrs	16–18 yrs
	Both	Boys	Girls	Boys	Girls	Girls	Boys	Girls
Swarna Vasundhara								
Energy	10.82	8.65	6.63	7.14	5.14	6.13	4.43	5.88
Protein	71.38	49.65	35.69	34.61	25.38	26.56	20.76	24.83
Fat	27.40	22.83	19.57	19.57	15.22	17.13	13.70	19.57
Dietary fiber	26.45	20.35	16.03	17.63	12.30	14.69	10.58	13.92
calcium	22.73	19.23	14.71	14.71	12.50	12.50	11.91	11.91
iron	32.73	24.00	22.50	12.86	16.36	12.00	13.85	11.25
zinc	30.00	22.88	15.88	15.88	9.44	10.55	7.67	9.51
Magnesium	54.99	39.28	28.64	27.50	19.92	20.22	15.62	18.09
Phosphorus	18.39	16.09	12.87	12.87	10.73	10.73	10.73	10.73
Karune								
Energy	11.58	9.26	7.09	7.64	5.51	6.56	4.74	6.30
Protein	95.56	66.48	47.78	46.33	33.98	35.56	27.80	33.24
Fat	27.56	22.97	19.69	19.69	15.31	17.23	13.78	19.69
Dietary fiber	26.40	20.31	16.00	17.60	12.28	14.67	10.56	13.89
Calcium	22.39	18.94	14.48	14.48	12.31	12.31	11.73	11.73
Iron	42.09	30.87	28.94	16.54	21.05	15.43	17.81	14.47
Zinc	30.44	23.22	16.12	16.12	9.58	10.70	7.78	9.65
Magnesium	62.01	44.29	32.30	31.00	22.47	22.80	17.62	20.40
Phosphorus	18.20	15.93	12.74	12.74	10.62	10.62	10.62	10.62
AGS447								
Energy	10.14	8.11	6.21	6.70	4.82	5.75	4.15	5.52
Protein	78.81	54.83	39.41	38.21	28.02	29.33	22.93	27.41
Fat	23.80	19.83	17.00	17.00	13.22	14.88	11.90	17.00
Dietary fiber	28.80	22.15	17.45	19.20	13.40	16.00	11.52	15.16
Calcium	21.81	18.46	14.11	14.11	12.00	12.00	11.42	11.42
Iron	39.36	28.87	27.06	15.46	19.68	14.43	16.65	13.53
Zinc	32.67	24.92	17.29	17.29	10.28	11.48	8.35	10.35
Magnesium	57.86	41.33	30.14	28.93	20.97	21.27	16.44	19.03
Phosphorus	15.52	13.58	10.87	10.87	9.06	9.06	9.06	9.06

**Table 11:** Percentage adequacy of RDA met by the consumption of vegetable soybean genotypes at the R6 stage in different age groups of children (30 g serving size).

Nutrients	4–6 yrs	7–9 yrs	10–12 yr	10–12 yr	13–15 yrs	13–15 yrs	16–18 yrs	16–18 yrs
	Both	Both	Boys	Girls	Boys	Girls	Boys	Girls
AVSB2001								
Energy	10.27	8.21	6.29	6.78	4.88	5.82	4.21	5.59
Protein	60.28	41.93	30.14	29.23	21.43	22.43	17.54	20.97
Fat	26.66	22.22	19.05	19.05	14.81	16.67	13.33	19.05
Dietary fiber	28.16	21.66	17.06	18.77	13.10	15.64	11.26	14.82
Calcium	19.42	16.44	12.57	12.57	10.68	10.68	10.17	10.17
Iron	32.15	23.58	22.11	12.63	16.08	11.79	13.60	11.05
Zinc	24.20	18.46	12.81	12.81	7.62	8.51	6.19	7.67
Magnesium	51.57	36.83	26.86	25.78	18.68	18.96	14.65	16.96
Phosphorus	9.37	8.19	6.56	6.56	5.46	5.46	5.46	5.46
AVSB2004								
Energy	10.17	8.13	6.23	6.71	4.83	5.76	4.16	5.53
Protein	56.70	39.44	28.35	27.49	20.16	21.10	16.49	19.72
Fat	25.98	21.65	18.56	18.56	14.43	16.24	12.99	18.56
Dietary fiber	27.72	21.32	16.80	18.48	12.89	15.40	11.09	14.59
Calcium	19.29	16.32	12.48	12.48	10.61	10.61	10.10	10.10
Iron	28.55	20.94	19.63	11.22	14.28	10.47	12.08	9.82
Zinc	22.13	16.88	11.72	11.72	6.97	7.78	5.66	7.01
Magnesium	49.08	35.06	25.57	24.54	17.78	18.05	13.94	16.15
Phosphorus	10.26	8.98	7.18	7.18	5.99	5.99	5.99	5.99
AGS447								
Energy	10.30	8.24	6.31	6.80	4.90	5.83	4.22	5.60
Protein	67.03	46.63	33.52	32.50	23.83	24.94	19.50	23.32
Fat	26.86	22.38	19.18	19.18	14.92	16.79	13.43	19.18
Dietary fiber	32.61	25.08	19.76	21.74	15.17	18.12	13.04	17.16
Calcium	15.80	13.37	10.22	10.22	8.69	8.69	8.28	8.28
Iron	33.25	24.38	22.86	13.06	16.62	12.19	14.07	11.43
Zinc	20.47	15.61	10.84	10.84	6.44	7.20	5.23	6.49
Magnesium	62.65	44.75	32.63	31.32	22.70	23.03	17.80	20.61
Phosphorus	11.15	9.75	7.80	7.80	6.50	6.50	6.50	6.50

## 4. Conclusions

Seed nutritional quality is fundamentally determined by the metabolic composition at various developmental stages, making detailed knowledge of seed chemistry crucial for advancing modern agricultural breeding strategies. With the advent of technologies such as metabolic engineering, precise characterization of seed metabolites enables targeted improvement of seed yield and nutrient profiles. Vegetable soybean, recognized for its agronomic significance and superior nutritional attributes, presents an excellent model for such advancements. This study highlights the critical importance of the R6 and R8 developmental stages in defining seed nutritional quality and sensory acceptability. The R6 stage, which is the early reproductive stage, is crucial as it is when the seeds start accumulating nutrients and is a key period for nutrient uptake. The R8 stage, which is the full maturity stage, is also significant as this is when the seeds reach their maximum nutrient content. At the R6 stage, genotypes Swarna Vasundhara, Karune, and AGS447 exhibited markedly superior nutritional profiles, characterized by high protein content, low fat levels, and rich concentrations of essential micronutrients coupled with enhanced in vitro protein digestibility. These attributes position the R6 stage seeds as valuable sources of nutrient-dense, health-promoting food ingredients. The R8 stage genotypes, while representing a later maturity phase, also demonstrated significant nutritional potential, contributing appreciable levels of protein, beneficial fats, dietary fiber, and minerals.

Understanding these stage-specific nutrient dynamics is essential for producers to optimize harvest timing, thereby maximizing vegetable soybean crops’ nutritional value and marketability. For food processors, knowledge of these compositional variations supports strategic decisions on ingredient selection and product development, particularly when utilizing vegetable soybeans as fortifying agents or functional components to enhance the nutritional quality of food formulations. End-users benefit from including vegetable soybeans in their diets as a sustainable source of high-quality protein and micronutrients, which is particularly vital in regions burdened by protein–energy malnutrition and micronutrient deficiencies. Moreover, cultivating short-duration vegetable soybean varieties aligns with sustainable agricultural practices, offering an ecologically viable pathway to enhance nutritional security while promoting local economies and livelihoods. The insights from this research thus provide a valuable foundation for breeders, who play a crucial role in developing specialty soybean cultivars tailored to specific food applications and nutritional needs. This empowers producers and processors to optimize crop utilization for improved health outcomes, highlighting the integral role of breeders in the process.

## Data Availability

The data presented in this study are available on request from the corresponding author.
